# Abundance, localization, and functional correlates of the advanced glycation end‐product carboxymethyl lysine in human myocardium

**DOI:** 10.14814/phy2.13462

**Published:** 2017-10-25

**Authors:** Martin M. LeWinter, Douglas Taatjes, Takamaru Ashikaga, Bradley Palmer, Nicole Bishop, Peter VanBuren, Stephen Bell, Cameron Donaldson, Markus Meyer, Kenneth B. Margulies, Margaret Redfield, David A. Bull, Michael Zile

**Affiliations:** ^1^ Cardiology Unit University of Vermont College of Medicine Burlington Vermont; ^2^ NHLBI Heart Failure Research Network Bethesda Maryland; ^3^ Cardiology Division Medical University of South Carolina Charleston South Carolina

**Keywords:** Advanced glycation end‐products, carboxymethyl lysine, diabetes mellitus, hypertension, myocardium

## Abstract

Advanced glycation end‐products (AGEs) play a role in the pathophysiology of diabetes mellitus (DM) and possibly hypertension (HTN). In *experimental* DM, AGEs accumulate in myocardium. Little is known about AGEs in *human* myocardium. We quantified abundance, localization, and functional correlates of the AGE carboxymethyl lysine (CML) in left ventricular (LV) myocardium from patients undergoing coronary bypass grafting (CBG). Immunoelectron microscopy was used to quantify CML in epicardial biopsies from 98 patients (71 M, 27 F) with HTN, HTN + DM or neither (controls), all with normal LV ejection fraction. Myofilament contraction‐relaxation function was measured in demembranated myocardial strips. Echocardiography was used to quantify LV structure and function. We found that CML was abundant within cardiomyocytes, but minimally associated with extracellular collagen. CML counts/*μ*m^2^ were 14.7% higher in mitochondria than the rest of the cytoplasm (*P* < 0.001). There were no significant sex or diagnostic group differences in CML counts [controls 45.6 ± 3.6/*μ*m^2^ (±SEM), HTN 45.8 ± 3.6/*μ*m^2^, HTN + DM 49.3 ± 6.2/*μ*m^2^; *P* = 0.85] and no significant correlations between CML counts and age, HgbA1c or myofilament function indexes. However, left atrial volume was significantly correlated with CML counts (*r* = 0.41, *P* = 0.004). We conclude that in CBG patients CML is abundant within cardiomyocytes but minimally associated with collagen, suggesting that AGEs do not *directly* modify the stiffness of myocardial collagen. Coexistent HTN or HTN + DM do not significantly influence CML abundance. The correlation of CML counts with LAV suggests an influence on diastolic function independent of HTN, DM or sex whose mechanism remains to be determined.

## Introduction

Advanced glycation end‐products (AGEs) result from adduction of glucose to proteins with subsequent modification by a series of nonenzymatic oxidation‐reduction reactions (Singh et al. 2001; Goldin et al. 2006; Ramasanmy and Schmidt 2012; Ward et al. 2013; Bodiga et al. 2014). AGEs contribute to the pathophysiology of diabetes mellitus (DM) (Singh et al. 2001; Goldin et al. 2006; Ramasanmy and Schmidt 2012; Ward et al. 2013; Bodiga et al. 2014) and are also considered to play a role in hypertension (HTN) (Kass et al. 2001; Zieman et al. 2007) and atherosclerosis (Chen et al. 2010; Campbell et al. 2011). From a functional standpoint, AGEs may alter ion transport (Bodiga et al. 2014; Yan et al. 2014; Yuan et al. 2014) and energy metabolism (Ward et al. 2013; Nelson et al. 2015) by modification of proteins involved in these processes. In collagen AGEs can cross‐link adjacent fibrils (Kass et al. 2001; Candido et al. 2003; Reddy 2004; Haus et al. 2007; Ramasanmy and Schmidt 2012), increasing its stiffness. In addition, activation of the receptor for AGEs (RAGE) in vascular endothelium elicits proinflammatory, profibrotic signaling resulting in increased collagen content (Singh et al. 2001; Goldin et al. 2006; Zieman et al. 2007; Ramasanmy and Schmidt 2012; Ward et al. 2013; Bodiga et al. 2014; Ott et al. 2014). In patients, skin and plasma levels of AGEs are correlated with left ventricular (LV) diastolic dysfunction (Hartog et al. 2008; Willemsen et al. 2011; Campbell et al. 2012). Elevated plasma levels increase the risk of heart failure (HF) (Hartog et al. 2007; Koyama et al. 2007).

Little is known about the abundance and localization of AGEs in *human* myocardium, their relationship with conditions such as DM and HTN, and their functional consequences. Carboxymethyl‐lysine (CML) is a ubiquitous AGE linked to various complications of DM (Schleicher et al. 1997; Amin et al. 2011; Choudhuri et al. 2013; Llauradó et al. 2014; Mishra et al. 2015). It is also a key ligand for RAGE (Xue et al. 2011). In light microscopic studies employing a CML‐specific antibody in LV endomyocardial biopsies (Schalkwijk et al. 2004; van Heerebeek et al. 2008; Falcão‐Pires et al. 2011) from patients with and without HF, CML was detected in small blood vessels but not in extracellular matrix (ECM) or in the cytoplasm of cardiomyocytes and was more abundant in patients with DM. Campbell et al. (2011) employed the same antibody to study immunolocalization by light microscopy in LV epicardial biopsy specimens obtained in the operating room from patients with coronary artery disease (CAD) undergoing coronary bypass grafting (CBG). They also detected CML exclusively in small blood vessels but its abundance did not differ amongst patients with type 2 DM, metabolic syndrome and those without these diagnoses. (Nożyński et al. 2009, 2013, 2012) employed light microscopic immunolocalization using anti‐AGE‐horseradish peroxidase antibodies in end‐stage failing hearts from patients with and without DM and nonfailing controls. In contrast to prior reports (Schalkwijk et al. 2004; van Heerebeek et al. 2008; Falcão‐Pires et al. 2011) they observed AGEs within cardiomyocytes but did not comment on ECM localization. They also reported that AGEs were more abundant in patients with DM. The specificities of their antibodies were not described.

We recently reported an *immunoelectron* microscopic (IEM) method to detect CML in myocardium using a specific antibody (Donaldson et al. 2010). Antigen‐antibody complexes can be identified with much higher resolution than light microscopy and quantified per unit area. In our preliminary paper (Donaldson et al. 2010) using epicardial biopsies from CBG patients CML was abundant in the cytoplasm of cardiomyocytes but association with ECM collagen was limited. However, the number of patients in this report was small, all but one was male, and we did not evaluate the relationship between CML abundance and functional consequences or links to a systemic proinflammatory/profibrotic state.

In this study we used the IEM method to test the hypothesis that HTN and combined HTN and DM are associated with increased CML abundance and to delineate CML localization in a much larger group of male and female CBG patients with normal LV ejection fraction (EF) divided into three groups: (1) controls without HTN or DM; (2) HTN; and (3) HTN+DM. We related CML measurements to clinical and demographic variables, echocardiographic measures of LV structure‐function, contraction‐relaxation properties of demembranated (skinned) strips obtained from the biopsies, and a panel of proinflammatory/profibrotic plasma biomarkers. We also measured myocardial CML in a group of brain‐dead organ donors without known heart or coronary artery disease (CAD). Finally, in a small subset we used the IEM method to examine pentosidine, an AGE that undergoes extensive cross‐linking (Sims et al. 1996; Reddy 2004; Haus et al. 2007; Avery et al. 2009).

## Methods

### Patient population

We recruited 71 male and 27 females for intraoperative myocardial biopsy from amongst those scheduled for CBG at (1) University of Vermont Medical Center (UVMMC), Burlington, Vermont, the clinical facility of the UVM College of Medicine (UVMCOM); (2) the Ralph H. Johnson VAMC and Medical University of South Carolina Hospital Authority, Charleston, South Carolina (referred to collectively as MUSC); and (3) selected NHLBI Heart Failure Research Network (HFN) Centers [University of Alberta (Alberta, Canada), Intermountain Medical Center (Murray, UT), Mayo Clinic (Rochester, MN), Minnesota Heart Institute (Minneapolis, MN), University of Utah and Utah VA Medical Center (Salt Lake City, UT)] between October 1, 2008 and August 6, 2012. All patients signed consent forms approved by their respective IRBs. Most of these patients also were enrolled in a previously published report on passive myocardial stiffness in HFpEF (Zile et al. 2015).

Preoperative research echocardiograms were performed in patients recruited at UVMMC and MUSC. Skinned strip studies described below were performed at UVMCOM using tissue from all patients recruited at these sites. A panel of selected serum biomarkers was measured in UVMMC and MUSC patients. At HFN centers, patients were purposely recruited who are relatively uncommon among the typical CBG population, specifically, females and controls of both sexes. HFN patients met LVEF inclusion criteria based on local echocardiograms or, in a few cases, LV cineangiograms, but did not have research echocardiograms or biomarker determinations. Skinned strip studies were performed in tissue from HFN sites if biopsies were sufficient in size and arrived at UVMCOM within 4 days of being obtained.

Patients were enrolled who met the following inclusion criteria: (1) age > 21 years; (2) LVEF > 0.50; (3) normal anterior and lateral wall motion during preoperative assessment by echocardiography or LV cineangiography (at HFN sites, we used the local site clinical interpretation of echocardiograms and ventriculograms for this purpose).

Patients were excluded for the following reasons: (1) Previous transmural myocardial infarction; (2) significant valvular or non‐coronary heart disease; (3) chronic pulmonary disease requiring oxygen therapy; (4) non‐ cardiac diseases or conditions that can affect myocardial function; (5) anemia (Hgb < 13.0 g/dl); (6) serum creatinine > 2.0 mg/dL; (7) off‐pump or emergency CBG; (8) active malignancy, infection, connective tissue disease, severe liver disease; (9) inability to provide informed consent.

HTN was diagnosed if patients had been told of the diagnosis and/or it was documented in their medical records *and* they were receiving medications to lower their blood pressure. DM was diagnosed if documented in the medical records. Patients were classified as controls if they had neither HTN nor DM.

Demographic, historical and laboratory data, medications and coronary anatomy were recorded. CAD severity was graded based on number of major vessels (left anterior descending and circumflex, right coronary artery) with a stenosis >70%, with left main coronary artery considered as two vessels.

Hearts from brain‐dead organ donor subjects were made available through the Gift of Life Donor Program (Philadelphia, PA). Patients thought to have died of cardiac disease or to have CAD in life were excluded. After in situ cardioplegia, hearts were explanted and transported on ice to the laboratory of K.B.M. at the University of Pennsylvania. Full thickness biopsies of the anterior LV were obtained from 6 hearts from eligible patients. ~ 1 mm^3^ specimens were dissected from epicardial, midwall, and endocardial layers and underwent CML analysis.

### Echocardiography

Echocardiograms obtained at UVMMC and MUSC were interpreted in blinded fashion at MUSC by MZ using standard criteria (Zile et al. 2015). Measurements included LVEF, end‐diastolic volume index (EDVI), relative wall thickness (RWT), left atrial (LA) volume, E/E' and assessment of regional wall motion.

### Myocardial biopsy procedure

Anterior LV free wall subepicardial biopsies weighing ~25–50 mg were obtained during CBG as previously described (Donaldson et al. 2012; Zile et al. 2015). No adverse effects ascribable to the biopsy were encountered. Biopsies were first placed in HEPES‐based Krebs solution (Donaldson et al. 2012; Yan et al. 2014). An ~1 × 1 mm sample was removed and processed for CML analysis (Donaldson et al. 2010). The rest of the tissue was dissected into strips and placed in skinning solution at 4°C. For MUSC and HFN samples, skinning coincided with transportation to UVMCOM. After skinning, strips were sculpted to 150–200 *μ*m diameter and 800–1200 *μ*m length, stored at −20°C and studied within 1 week of being obtained. We have previously shown (Donaldson et al. 2012) that key myofilament proteins are not degraded when stored in this fashion.

### AGE analysis

CML antigen‐antibody complexes were counted/*μ*m^2^ in the cytoplasm and within mitochondria (Donaldson et al. 2010). We discontinued mitochondrial counts when an interim analysis revealed a highly significant difference between cytoplasm and mitochondria. We applied the same method using an antibody to pentosidine (Biologo, Kronshagen, Germany; #PEN012, mouse IgG1) in four control patients.

### Myofilament function

These methods have been described previously (Donaldson et al. 2012; Zile et al. 2015). Measurements were made at sarcomere length 2.2 *μ*m. Steady‐state isometric force was measured at pCa 8 and then as pCa was gradually decreased to 4.5. Tension (T) was calculated as force per cross‐sectional area. T_MAX_ was taken at pCa 4.5. T minus relaxed tension at pCa 8 (T_pCa8_) at each pCa was normalized to maximum developed tension (T_MAX_–T_pCa8_). These data were used to estimate pCa_50_. Passive tension was taken as T_pCa8_.

### Plasma biomarkers

Biomarkers reflecting ECM homeostasis [matrix metalloproteinases (MMPs) −1,−2, −3, −7, −8 and −9 and tissue inhibitors (TIMPs) −1, −2, −3, and −4], and a proinflammatory/profibrotic state [C‐reactive protein (CRP), interleukin 6 (IL‐6), IL‐8, tumor necrosis factor‐*α* (TNF‐*α*), and soluble ST2 (sST2)] were measured as well as NT‐proBNP) (Zile et al. 2015). HgbA1c was measured in the clinical laboratory in UVMMC/MUSC DM patients.

### Statistical analysis

Data are presented as mean ± SEM. Descriptive statistics including Pearson correlation coefficients were obtained for all subjects by diagnostic groups. Chi square contingency tables were used to compare gender and drug use across diagnostic groups followed by paired comparisons using Fisher's Exact Test only if significant overall differences were observed. Within subject comparisons of cytoplasmic and mitochondrial CML counts were conducted using paired t‐tests overall and for each diagnostic group. Comparisons of CML counts and other measures between diagnostic groups were performed using one‐way ANOVA while gender was incorporated in two‐way ANOVA models. Statistically significant ANOVA results were followed by paired comparisons using Fisher's Least Significant Differences (LSD) approach. *P* ≤ 0.05 was considered statistically significant.

## Results

A total of 98 patients were enrolled (34 control, 34 HTN, and 30 HTN + DM). Mean age, body mass index (BMI), serum creatinine, and drug usage are shown in Table 1. For BMI, there was a significant group difference (*P* = 0.005), with BMI greater in HTN + DM than controls (*P* < 0.001). There were no significant differences by sex for any of these variables. As shown in Table 1, there were significant group differences in the use of beta‐blockers, ACEIs/ARBs and other blood pressure lowering drugs, but not statins. A total of 28/30 HTN + DM patients were receiving hypoglycemic agents (19/30 insulin, 18/30 other agents). Mean HgbA1c was 7.4 ± 0.5% (*n* = 24). The number of vessels with stenoses > 70% was 2.5 ± 0.12 in the control and HTN groups and 2.5 ± 0.13 in the HTN + DM group.

**Table 1:** Clinical data and drug use (% receiving drug)

	Controls (*n *= 34)	HTN (*n* = 34)	HTN + DM (*n* = 30)	*P*‐value
Age, years (±SD)	66.3 ± 1.4	67.1 ± 1.7	62.2 ± 2.6	0.173^a^
BMI, kg/m^2^	27.3 ± 0.5	29.5 ± 1.1	31.6 ± 1.0^c^	0.005
Creatinine, mL/dL	0.98 ± 0.03	0.99 ± 0.05	1.00 ± 0.05	0.946
Drug use				
* β*‐blockers	12 (35%)	22 (65%)	27 (90%)^d^	<0.001^b^
* *ACEIs/ARBs	2 (6%)	21 (62%)	22 (73%)^e^	<0.001
* *Other BP drugs 0 (0%)	0 (0%)	10 (29%)	6 (20%)^f^	0.005
* *Statins	20 (59%)	25 (74%)	25 (83%)	0.091

a Analysis of Variance followed by Fisher Least Significant Difference Tests.

b 2x3 Pearson Chi‐Square followed by Fisher's Exact Test.

c HTN + DM versus Control (*P* = 0.001).

d HTN + DM versus Control (*P* < 0.001) versus HTN (*P* = 0.021) and HTN versus Control (*P* = 0.028).

e HTN + DM versus Control (*P* < 0.001) and HTN versus Control (*P* < 0.001).

f HTN + DM versus Control (*P* = 0.008) and HTN versus Control (*P* = 0.001).

### AGE abundance and localization

**Figure 1:**
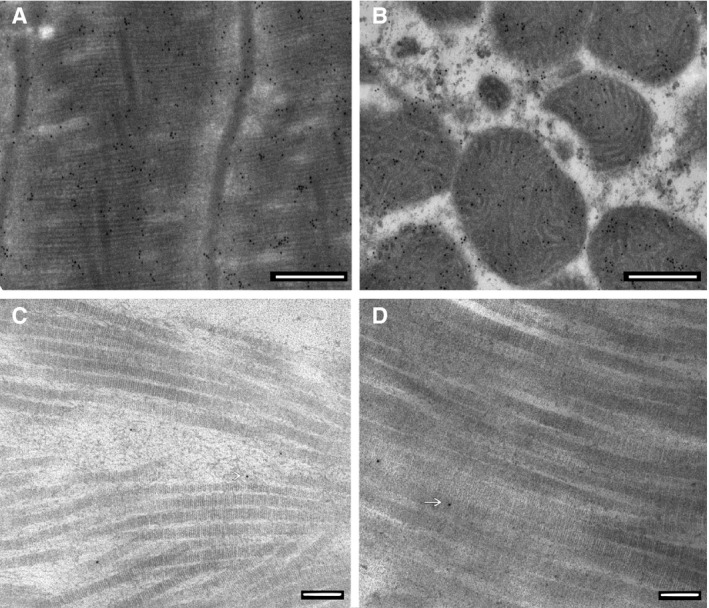
Panel A, cytoplasmic images of CML antigen‐antibody complexes (black dots) from a representative CBG patient (Magnification = 8000X); panel B, mitochondrial images from the same patient (Magnification = 8000X); panel C, ECM collagen images using CML antibody in a representative CBG patient (Magnification = 30,000X); panel D, ECM collagen images using pentosidine antibody (Magnification = 30,000X). In panels C and D, the arrows point to one of the few identifiable antigen‐antibody complexes.

As shown in Figure 1, panels A and B, CML antigen‐antibody complexes (seen as discrete black dots) were abundant within the cytoplasm and mitochondria of cardiomyocytes. There was no obvious localization pattern except that counts were 14.7% higher within mitochondria compared with cytoplasm (52.2 ± 4.6/*μ*m^2^ vs. 45.5 ± 4.2/*μ*m^2^, *n* = 57, *P* < 0.001). Differences between mitochondrial and cytoplasmic counts were similar in each group (11.0% controls, 13.8% HTN, 17.0% HTN + DM). Subsequently, we report cytoplasmic counts only. There were no significant differences in CML counts by sex for the entire cohort (*P* = 0.34) and no significant interaction across the three diagnostic groups by sex (ANOVA *P* = 0.82). Therefore, sexes were combined for analyses of counts within each group. There were no significant differences in CML counts among the three diagnostic groups (ANOVA *P* = 0.85). Mean values were 45.6 ± 3.6/*μ*m^2^ in controls, 45.8 ± 3.6/*μ*m^2^ in HTN, and 49.3 ± 6.2/*μ*m^2^ in HTN + DM.

Localization of CML in association with ECM collagen fibrils was minimal (Figure 1, panel C). Because of the scarcity of ECM CML and the fact that the number of fibrils within each field was variable we did not attempt to quantify ECM counts. Pentosidine, an AGE known to extensively cross‐link (Sims et al. 1996; Reddy 2004; Haus et al. 2007; Avery et al. 2009), was analyzed in four controls. Results were qualitatively indistinguishable from CML, that is, pentosidine was abundant in cytoplasm but minimally associated with collagen (Figure 1, panel D).

There were no significant correlations between cytoplasmic CML counts and age in the complete sample (*P* = 0.84) and within each group. There were no significant correlations between CML and BMI in the complete sample (*P* = 0.53) and in HTN and HTN+DM groups (*P* = 0.91 and 0.92, respectively). However, we found a positive correlation between CML counts and BMI in controls (*r* = 0.57, *P* = 0.04). There was no significant correlation between CML and HgbA1c in HTN + DM patients (*r* = 0.23, *P* = 0.27).

ACEIs/ARBs, statins and hypoglycemic agents are reported to reduce AGE accumulation (Calkin et al. 2008; Chen et al. 2009; Lenski et al. 2011; Ishibashi et al. 2012; Mirmiranpour et al. 2013; Tian et al. 2014; Romero et al. 2015). All HTN + DM patients were receiving at least one and most were receiving three of these drugs (Table 1). Thus, there were no untreated HTN + DM patients available to test for potential drug treatment effects. However, 6 HTN patients were not receiving any of these drugs. Therefore, to test for treatment effects we compared CML counts in these 6 to the other HTN patients. In addition, to test for effects of HTN per se we also compared the 6 untreated HTN patients with 13 controls who were not receiving any of these drugs. CML counts were similar in each of these groups [untreated HTN 47.8 ± 15.9/*μ*m^2^, treated HTN 45.3 ± 4.8/*μ*m^2^, untreated controls 47.7 ± 7.5/ *μ*m^2^ (*P* = 0.85 vs. treated HTN, 1.0 vs. untreated controls)].

**Figure 2:**
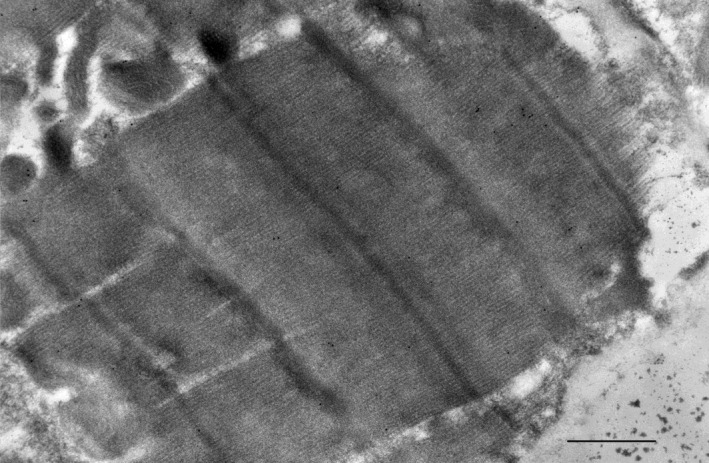
Images from a brain dead patient showing small numbers of CML antigen‐antibody complexes in cytoplasm of a cardiomyocyte (Magnification = 8000X).

Clinical characteristics and CML counts of the 6 donor patients without known heart disease are shown in Table 2. They were evenly distributed by sex and varied widely in age. None were known to have DM. One (#1436) had a diagnosis of HTN and may have been receiving lisinopril. Other than pressors, no other patient was known to be receiving cardiovascular drugs. Three of the six patients (#1400, #1406, #1407) had normal coronary angiograms. In four of the six donor patients CML counts were in single digits. Counts in these patients were lower than those in *any* of the CBG patients, the lowest of whom had a value of 14/*μ*m^2^ (Fig. 2). The other two brain dead patients (who did *not* undergo coronary angiography) had CML counts in the same range as CBG patients. There were no obvious differences in epicardial, midwall and endocardial CML counts and, as in CBG patients, counts were higher in mitochondria than the rest of the cytoplasm (Table 2). Similar to the CBG patients, CML antigen‐antibody complexes were very sparse in the ECM in any layer of the myocardium.

**Table 2:** Clinical characteristics and CML counts for brain dead patients Counts (per *μ* ^2^)

Case number	Age	Sex	Diagnoses	Cytoplasm			Mitochondria		
				Epi	Mid	Endo	Epi	Mid	Endo
1400	22	F	CNS bleed	5	4	3	7	9	6
1406	51	M	CNS bleed	5	9	9	8	23	14
1407	31	M	Sepsis	5	7	7	8	11	9
1414	74	M	CNS bleed	3	4	3	3	6	5
1436	57	F	Drug overdose	53	53	71	63	63	81
1440	74	F	Head trauma	63	62	57	74	65	67

### Echocardiography‐doppler studies

Echocardiographic‐Doppler data are shown in Table 3, with sexes grouped together. There were no significant group differences in EF or end‐diastolic volume index (EDVI)**.** However, there were significant differences between diagnostic groups in RWT (*P* = 0.01) and LA volume (*P* = 0.002). Individual testing revealed a higher RWT in HTN (*P* = 0.03) and HTN+DM (*P* = 0.003) versus controls. LA volume was significantly greater in HTN versus controls (*P* = 0.001), with a trend toward a higher value in HTN+DM (*P* = 0.052). There was also a trend toward a difference (*P* = 0.06) between diagnostic groups for E/E', with higher values in HTN and HTN+DM. The only significant correlations with CML counts were LA volume in the HTN group (*r* = 0.62, *P* = 0.019) and LA volume for the entire cohort (*r* = 0.41, *P* = 0.004).

**Table 3:** Echocardiography results

	Controls (*n* = 14)	HTN (*n* = 20)	HTN+DM (*n* = 22)	*P*–value^c^
EF (%)	66 ± 1.8	63 ± 2.5	65 ± 1.6	0.582
EDVI (mL /m^2^)	114 ± 6.1	127 ± 6.4	130 ± 5.2	0.184
RWT	0.39 ± 0.01	44 ± 0.02^a^	0.46 ± 0.01^a^	0.010
LA Volume (mL) 20.6 ± 1.0	20.6 ± 1.0	37.1 ± 4.0^b^	29.0 ± 2.1	0.002
E/E'	7.9 ± 0.60	8.5 ± 0.80	10.7 ± 0.89	0.061

EF, LV ejection fraction; EDVI, LV end‐diastolic volume index; RWT, relative wall thickness; LA, left atrium.

a HTN + DM versus Control (*P* = 0.003) and HTN versus Control (*P* = 0.030).

b HTN versus Control (*P* = 0.001).

c Analysis of variance followed by Fisher's Least Significant Difference Tests.

### Myofilament function

There was a significant diagnostic group difference for T_MAX_ (*P* = 0.024). Individual testing revealed a higher value in controls (19.0 ± 1.1 mN·mm^−2^) versus HTN (12.1 ± 0.8 mN·mm^−2^, *P* = 0.011) but not HTN+DM (18.1 ± 1.6 mN·mm^−2^, *P* = 0.70). There were no group differences in pCa_50_ or T_pCa8_ and no significant correlations with CML counts in any group or the entire cohort.

### Plasma biomarkers

There were significant group differences in TIMP1 (*P* < 0.001), CRP (*P* = 0.006), and ST2 (*P* = 0.002) (Table 4). By individual testing, TIMP1 was greater in HTN (*P* = 0.008) and HTN+DM (*P* < 0.001) versus controls. For CRP, HTN was greater than controls (*P* = 0.001). ST2 was significantly greater in HTN (*P* < 0.001) and HTN+DM (*P* = 0.006) versus controls. There was a trend toward a difference for NT pro‐BNP by diagnostic group (*P* = 0.084), with higher values in HTN and HTN + DM compared with controls. No other biomarkers achieved statistical significance for group differences (all *P* > 0.18). The only significant CML‐biomarker correlation was with TIMP1 in controls (*r* = −0.76, *P* = 0.016).

**Table 4:** Selected biomarkers

	Controls (*n* = 11)	HTN (*n* = 14)	HTN + DM (*n* = 19)	*P* value^d^
TIMP1 (ng/mL)	63 ± 8.6	106 ± 7.4	124 ± 10.1^a^	<0.001
CRP (*μ*g/mL)	5.4 ± 0.7	5.4 ± 0.7	3.7 ± 0.57^b^	0.006
ST2 (ng/mL)	27 ± 6.1	102 ± 12.5	80 ± 13.6^c^	0.002
NT pro‐BNP (pg/mL)	299 ± 79	825 ± 157	733 ± 112	0.084

a HTN + DM versus Control (*P* < 0.001) and HTN versus Control (*P *= 0.008).

b HTN + DM versus Control (*P* = 0.001) and HTN + DM versus HTN (*P* = 0.043).

c HTN + DM versus Control (*P* = 0.006) and HTN versus Control (*P* < 0.001).

d Analysis of Variance followed by Fisher's Least Significant Difference Tests.

## Discussion

Using an electron microscopic method that provides much greater resolution than light microscopy (Donaldson et al. 2010), we found that CML is abundant in the cytoplasm of cardiomyocytes from CBG patients. Abundance was not affected by coexistent HTN or HTN + DM. CML counts were positively correlated with BMI in controls, but not HTN or HTN + DM patients, suggesting that BMI is unmasked as a driver for AGEs in the absence of HTN or DM. CML appeared to be distributed randomly within the cytoplasm, except that counts were systematically somewhat higher within mitochondria than the rest of the cytoplasm. In contrast to the cytoplasm, localization in association with ECM collagen was very limited.

Previous reports of AGE distribution in human myocardium using *light* microscopy have been conflicting. Nożyński et al. (2009, 2013, 2012) detected CML *within* cardiomyocytes using antibodies whose specificity was not reported. Using a CML‐specific antibody, van Heerebeek et al. (2008), Schalkwijk et al. (2004), Falcão‐Pires et al. (2011) and Campbell et al. (2011) identified CML *only* within the endothelium of small blood vessels. We suspect that our ability to detect CML *within* cardiomyocytes, in contrast with the latter studies (Schalkwijk et al. 2004; van Heerebeek et al. 2008; Campbell et al. 2011; Falcão‐Pires et al. 2011), reflects the fact that IEM allows identification of individual antigen‐antibody complexes, whereas light microscopy does not have this level of resolution. Our review of images from these studies suggests that CML is detected in locations where multiple antigen‐antibody complexes occur in close proximity, rendering them more easily detectable by light microscopy.

In four of the six organ donor patients without obvious heart disease CML counts were lower than in *any* of the CBG patients. Two of the four with very low counts were 51 and 74 years old, that is, in the same age range as the CBG patients. While the number of organ donor patients was obviously small, these results demonstrate that CML abundance of the magnitude observed in CBG patients is not invariant. In the only report in which a wide age range was analyzed (Schleicher et al. 1997), CML was detected in adult tissues without age dependence. Higher mitochondrial counts were also evident in this group (Table 2). We speculate that the *uniformly* greater abundance of CML in CBG patients regardless of diagnostic group compared with four of the donor patients could be related to the systemic proinflammatory state present in CAD (Libby 2002; Libby et al. 2002), which may outweigh any effects of HTN and/or DM.

Cytoplasmic CML counts were similar in HTN + DM, HTN, and controls. In most prior light microscopic studies in human myocardium (Schalkwijk et al. 2004; van Heerebeek et al. 2008; Nożyński et al. (2009, 2013, 2012); Falcão‐Pires et al. 2011) a higher AGE abundance was reported in DM. However, these studies included large numbers of patients with HF and tissue was obtained from subendocardial biopsies performed in the cardiac catheterization laboratory. In a *light* microscopic study which was much more comparable to ours with respect to the nature of the patients and the biopsy method, Campbell et al. (Campbell et al. 2011) quantified CML in epicardial biopsies obtained in the operating room from controls and patients with type 2 DM and metabolic syndrome with normal EF undergoing CBG. They also did *not* detect group differences in CML abundance. One possibility accounting for the lack of significant differences in CML counts in controls, HTN and HTN + DM patients in our study is the fact that large numbers of HTN and HTN + DM patients were receiving ACEIs/ARBs and statins (Table 1), drugs with anti‐inflammatory and anti‐fibrotic properties that reduce AGE accumulation (Calkin et al. 2008; Ishibashi et al. 2012; Tian et al. 2014; Romero et al. 2015). While subgroup comparisons of untreated versus treated HTN patients and untreated controls failed to disclose differences consistent with a treatment or HTN effect, the number of untreated HTN patients available for analysis was small. In HTN + DM patients AGE accumulation may also have been reduced because virtually all were receiving hypoglycemic agents, which inhibit accumulation in animals and patients (Chen et al. 2009; Lenski et al. 2011; Ishibashi et al. 2012; Mirmiranpour et al. 2013).

In DM, AGEs are thought to increase tissue stiffness due to both collagen cross‐linking and RAGE activation, which causes increased collagen content (Candido et al. 2003; Goldin et al. 2006; Haus et al. 2007; Ramasanmy and Schmidt 2012; Bodiga et al. 2014; Ott et al. 2014). A similar role has been suggested in HTN (Chen et al. 2010; Campbell et al. 2011). Thus, it was surprising that CML localization in association with collagen was so limited. However, low abundance in ECM is consistent with the light microscopic studies cited earlier (Schalkwijk et al. 2004; van Heerebeek et al. 2008; Nożyński et al. 2009; Falcão‐Pires et al. 2011). Moreover, pentosidine results were qualitatively similar to our CML findings. Thus, an AGE thought to more readily cross‐link was also limited in abundance in the ECM. Several studies have tested whether the AGE cross‐link breaker alagebrium can improve diastolic stiffness. In normal rats (Jochen et al. 2012) and normal dogs (Mohammad et al. 2000), the drug was shown to partially reverse age‐associated increases in end‐diastolic chamber stiffness, results which implicate significant AGE cross‐linking in myocardial collagen as a function of aging. In contrast, in elderly hypertensive dogs (Shapiro et al. 2008) with markedly increased levels of vascular CML by light microscopic immunolocalization, alagebrium did not improve chamber stiffness, arguing against such a role. Importantly, studies in humans have also failed to demonstrate effects of alagebrium on diastolic function. Thus, the drug did not reverse age‐associated increases in noninvasively estimated myocardial stiffness in older normal subjects (Fujimoto et al. 2013), nor did it improve measures of diastolic function in patients with HFpEF (Little et al. 2005; Hartog et al. 2011). These previous human studies are thus very consistent with our finding of minimal ECM AGE accumulation and, hence, minimal AGE‐associated cross‐linking.

Virtually all DM patients were treated with hypoglycemic agents. In addition to contributing to lack of a difference in cytoplasmic CML in HTN + DM versus controls treatment might also reduce ECM accumulation. This could explain a discrepancy between our results and experimental DM, where AGEs *were* detected in the ECM in *untreated* hyperglycemia (Schafer et al. 2006). An alternative explanation for lack of AGE association with collagen is that multiple AGEs accumulate, each in relatively small amounts. However, this seems unlikely considering the ubiquity of CML and the fact that both CML and pentosidine were easily detectable within cardiomyocytes but not in ECM. Consistent with our findings in DM+HTN, in a recent study (Zile et al. 2015) we reported that patients with HFpEF undergoing CBG have increased passive myocardial stiffness and total and insoluble collagen (a marker of cross‐linking) compared with controls, but coexistent DM had no effect on these variables. As discussed above, our results argue against a major role for AGEs in increased collagen cross‐linking in CBG patients with or without DM or HTN, but does not exclude an effect on collagen *content* via RAGE. Collagen cross‐linking is normally carried out by lysyl oxidase (LOX) and a homologue, LOX‐like 2 (Yamauchi and Sricholpech 2012), which catalyze oxidation of lysine residues. Goldsmith et al. (2013) have proposed that LOX activity is increased under profibrotic conditions in the myocardium, suggesting a mechanism for non‐AGE dependent increased collagen cross‐linking in HFpEF.

Echocardiography revealed increased RWT in HTN and HTN + DM, indicating concentric remodeling. LA volume was larger in HTN versus controls and nearly so in HTN + DM (*P* = 0.052). E/E' was also nearly significantly greater in HTN and HTN + DM (ANOVA *P* = 0.06). Taken together, these results are consistent with diastolic dysfunction in association with concentric remodeling in HTN and HTN + DM. CML counts were positively correlated with LA volume in HTN patients and in the entire cohort, suggesting a direct relationship between CML and diastolic dysfunction. This correlation was highly significant. Moreover, LA volume is a relatively robust index of diastolic function as it is less load sensitive than Doppler‐derived indexes.

There were no correlations between CML and myofilament function, including pCa_50_, a determinant of relaxation rate, and resting tension (T_pCa8_). This suggests that the diastolic dysfunction we observed in HTN and HTN+DM patients does not have a *myofilament* basis. The limited amount of tissue available precluded analysis of other myocardial properties that influence diastolic function, for example, ion transport. Additionally, the relatively higher abundance of CML in mitochondria suggests the possibility of diastolic dysfunction caused by depressed energy‐dependent processes such as Ca^2+^ re‐uptake by the sarcoplasmic reticulum.

Biomarker correlations with CML counts were very modest. It is unclear why these were not more evident. Perhaps we did not select optimal biomarkers. However, we did observe diagnostic group biomarker differences and the biomarkers we chose are generally considered to provide good screening for alterations in inflammation and collagen homeostasis.

### Limitations

HFN sites recruited patients who are relatively scarce amongst the CBG population. This allowed us to quantify CML in larger numbers of females and controls. However, research echocardiograms and biomarkers were not obtained and myofilament studies could not be uniformly performed in these patients. Moreover, 98 patients is a relatively modest size cohort and relatively small group differences and/or correlations between CML counts and echocardiographic data, biomarkers and myofilament results could have been missed.

All of our CBG patient biopsies were obtained from the epicardial layer of the LV. Therefore, it is possible that transmural variations in CML or pentodisine abundance in cardiomyocytes and/or the ECM could have been missed. However, this seems somewhat unlikely since there were no obvious transmural CML differences in the donor hearts, including those with cytoplasmic CML counts similar to the CBG patients.

Another limitation is the possibility that some control patients had unrecognized hypertension, a common problem. However, we believe this is unlikely since all of the control patients had seen physicians at various times before CBG and had not been told of hypertension, none had elevated blood pressures during hospitalizations or clinic visits before CBG and none had received drugs to treat hypertension. Finally, we did not assess RAGE abundance in the biopsies. RAGE is a vascular endothelial receptor and it is in the nature of our biopsies that we do not consistently image small blood vessels. Had we been able to do so, this might have provided valuable additional information about the role of AGEs, for example as a cause of increased collagen content.

## Conclusions

CML is readily detectable within cardiomyocytes from CBG patients. Abundance is somewhat greater in mitochondria and not significantly influenced by sex or HTN with or without coexistent DM. Localization of CML and pentosidine in association with collagen is very limited. Taken together, our results do not support AGEs as an important mechanism of collagen cross‐linking and increased passive stiffness or of altered myofilament function in human myocardium. However, the highly significant correlation of cytoplasmic CML counts with LA volume suggests an as yet undefined role in diastolic dysfunction.

## Conflict of Interest

None declared.
